# Joint Torque Prediction via Hybrid Neuromusculoskeletal Modelling during Gait Using Statistical Ground Reaction Estimates: An Exploratory Study

**DOI:** 10.3390/s21196597

**Published:** 2021-10-02

**Authors:** Shui Kan Lam, Ivan Vujaklija

**Affiliations:** Department of Electrical Engineering and Automation, Aalto University, 02150 Espoo, Finland; ivan.vujaklija@aalto.fi

**Keywords:** joint torque, gait, neuromusculoskeletal modelling, inverse dynamics, machine learning, ground reaction force, centre of pressure

## Abstract

Joint torques of lower extremity are important clinical indicators of gait capability. This parameter can be quantified via hybrid neuromusculoskeletal modelling that combines electromyography-driven modelling and static optimisation. The simulations rely on kinematics and external force measurements, for example, ground reaction forces (GRF) and the corresponding centres of pressure (COP), which are conventionally acquired using force plates. This bulky equipment, however, hinders gait analysis in real-world environments. While this portability issue could potentially be solved by estimating the parameters through machine learning, the effect of the estimation errors on joint torque prediction with biomechanical models remains to be investigated. This study first estimated GRF and COP through feedforward artificial neural networks, and then leveraged them to predict lower-limb sagittal joint torques via (i) inverse dynamics and (ii) hybrid modelling. The approach was evaluated on five healthy subjects, individually. The predicted torques were validated with the measured torques, showing that hip was the most sensitive whereas ankle was the most resistive to the GRF/COP estimates for both models, with average metrics values being 0.70 < $R^{2}$ < 0.97 and 0.069 < RMSE < 0.15 (Nm/kg). This study demonstrated the feasibility of torque prediction based on personalised (neuro)musculoskeletal modelling using statistical ground reaction estimates, thus providing insights into potential real-world mobile joint torque quantification.

## 1. Introduction

Three-dimensional gait analysis aimed at quantifying joint and musculotendon kinetics is crucial for certain medical diagnosis, motor assessment and development of rehabilitative technologies [1,2,3]. Estimating joint moments through inverse dynamics modelling in gait anaylsis is regarded as the gold standard [4,5]. This approach solves Newton–Euler equations of motion for each rigid segment of a biomechanical model, beginning from the most distal one [6,7]. Then, inverse dynamics-based static optimisation or dynamic optimisation are often used to solve the redundancy problem of the musculoskeletal system in order to predict individual muscle forces [8,9,10]. Optimisation methods require definition of an objective function describing muscle recruitment strategies, which has remained a challenging endeavour. To this end, electromyography (EMG) observations are introduced to drive the model and estimate these kinetic parameters [11,12,13,14,15]. EMGs are electrical muscle signal recordings originating as output of motor neurons, and thus are informative of the neural drive to muscles [16]. Therefore, as opposed to optimisation-based approaches, EMG-driven modelling has the benefit of leveraging direct estimation of individual muscle contributions. Furthermore, neural control and musculoskeletal functions are inseparable components underlying human movements. Understanding the neuromuscular interlink is valuable for a wide range of purposes, such as designing rehabilitation therapies and studying gait pathology and disease prognostics [17,18,19]. Yet, our understanding of this neuromuscular relationship is still limited [20]. Hence, an added benefit of neuromusculoskeletal models is that they can help untangle the complexities of neural drive to muscles. However, this approach has its own shortcomings. EMG signals from deep muscles are difficult to record noninvasively, and cross-talk between muscles as well as movement artifacts can contaminate the signals and reduce the performance of the models [21,22]. In light of the limitations of both optimisation and EMG-driven methods, a hybrid model has been proposed to balance the uncertainties induced by the two approaches [23]. This model compares the joint torques predicted by an EMG-driven model and inverse dynamics, and minimises the torque differences by minimally fine-tuning the EMG signals and generating the excitations that have not been recorded.

In gait analysis, both inverse dynamics and the hybrid model rely on kinematics and external forces such as ground reaction force (GRF) and the corresponding centre of pressure (COP). Conventionally, these measurements are acquired using camera-based motion capture systems and force plates installed under the walkway, which as a concept poses some limitations. The size and weight of these instruments constitute the constraint of nonportability, causing real-world analyses to be almost infeasible. The high cost of the force plates further complicates analyses with a large number of consecutive gait cycles. To address the issues induced by force plates, past studies have attempted the use of pressure insoles and instrumented shoes [24,25,26,27,28]. This type of approach alleviates the problems and eases measurements outside the laboratory. Yet, the reliability of some insoles is still pending considerable advancement [29], and the size of the sensors attached beneath the shoes may cause discomfort to the users during gait, especially in long-distance investigations. Additionally, a certain kind of footwear could affect the measured GRF [30]. These outstanding challenges have motivated researchers to estimate GRF and COP through machine learning. Specifically, marker trajectories collected with motion capture systems and kinematics measured by such wearable sensors as inertial measurement units are employed to drive the statistical models for the estimation of the two gait parameters [31,32,33,34,35,36,37,38,39]. Despite that past literature has shown great potential of this approach to be used in real-world environments, there is still room for improvement in the design and application of statistical models incorporating GRF and COP estimation. The main concern here is the propagation of the resulting estimation errors through the bottom-up modelling process. Until now, the exact impact of these errors on joint torque prediction based on either musculoskeletal or neuromusculoskeletal models has yet to be investigated, and thus the feasibility of torque prediction relying on GRF and COP estimates remains unclear. 

Some previous studies have directly predicted joint torques via data-driven methods without relying on GRF or COP [33,37,39,40,41]. Their statistical models have been generalised across subjects, which is a useful tool for clinical analysis for specific target groups. The downside of this design is the incapability of capturing subject-specific musculoskeletal and neurophysiological characteristics, which is crucial for the development of personalised rehabilitation devices [20]. Furthermore, the black-box nature as well has its benefits and drawbacks; it does not require thorough understanding of complex intermediate biomechanical processes to obtain the final output, but at the same time, this does not provide insights into profound neuromusculoskeletal interaction.

The primary aim of this study is to explore the applicability of both the inverse dynamics and the hybrid neuromusculoskeletal model for sagittal joint torque prediction in less constraining environments. This implies limiting the use of force plates and relying on measurements that can eventually be obtained with fully wearable systems. We first estimated GRF and COP with machine learning algorithms, then used these estimates to support the prediction of joint torques via inverse dynamics and hybrid neuromusculoskeletal modelling. The ‘goodness-of-fit’ of this approach was evaluated by comparing the resulting torques with the conventionally obtained experimental ones. To the best of our knowledge, this is the first study to investigate joint moment prediction via (neuro-)musculoskeletal modelling with statistical GRF and COP estimates. The secondary goal here is then to examine the individual effect of GRF and COP estimation accuracy on the joint moments predicted by each of the two models. The main contribution of this study lies in exploring the paradigm that enhances the capabilities of translating personalised model-based joint torque quantification of this type from laboratories to daily-life environments.

## 2. Materials and Methods

### 2.1. Data Collection and Preprocessing

The study has been validated on two male and three female healthy subjects (35.2 ± 16.8 years, 1.71 ± 0.10 m, 71.0 ± 13.8 kg) from a public dataset recorded by Schreiber and Moissenet [42]. This dataset includes optical motion tracking, force-plate measurements and EMG recordings, which is regarded as a benchmark for our evaluation. Each of the subjects performed four trials of self-paced ground-level walking in an unconstrained fashion. For each trial, one complete stance phase of the right leg was analysed. The data collection protocol is briefly described here in accordance to the original work for the purpose of clarity [42]. Ground reaction forces and moments (GRFM) were collected at 1500 Hz with two force plates (OR6-5, AMTI, USA). Three-dimensional marker locations were recorded at 100 Hz using a 10-camera system (OQUS4, Qualisys, Sweden). EMG signals were measured at 1500 Hz (Desktop DTS, Noraxon, Scottsdale, AZ, USA) from eight right leg muscles: soleus, tibialis anterior, gastrocnemius medialis, rectus femoris, vastus medialis, semitendinosus, gluteus maximus and gluteus medius using bipolar surface electrodes (Ambu Neuroline 720, Ambu, Denmark).

Marker trajectories and GRFM were passed to zero-lag second-order Butterworth low pass filters with cutoff frequency of 8 Hz [43]. Marker locations were interpolated to the length of GRF. COP was calculated from GRFM. To generalise these measurements among subjects, heel marker position was taken as the reference. EMG data were high-pass filtered at 30 Hz, full-wave rectified, and then low-pass filtered at 6 Hz, with a zero-lag fourth-order Butterworth filter in order to obtain the EMG envelope [11]. The EMG envelopes were normalised with respect to the peak values across the entire set of data for each subject. Linear velocity and acceleration were calculated from the dynamic marker trajectories via frame-by-frame differentiation.

### 2.2. Musculoskeletal Modelling

In order to obtain individual joint torques, modelling analysis was performed using OpenSim [44]—an open-source biomechanical software system. A generic model presented by Hamner, Seth and Delp [45] was linearly scaled using 44 reflective markers to match the anthropometry of each subject. Tendon slack lengths and optimal fiber lengths of musculotendon units (MTUs) crossing hip, knee and ankle joints from the subject-specific model were then optimised as previously described in [46]. Corresponding joint angles were computed from dynamic marker trajectories via inverse kinematics. Angular velocity and acceleration were calculated from the joint angles via frame-by-frame differentiation. Musculotendon lengths and moment arms for hip and knee flexion/extension and ankle plantar/dorsi flexion were obtained with a muscle analysis tool. Joint torques for these degrees of freedom (DoF), referred to as ‘the measured torques’ hereafter, were computed via inverse dynamics.

### 2.3. EMG-Assisted Musculoskeletal Modeling

In contrast to inverse dynamics which only considers musculoskeletal aspects, the EMG-assisted musculoskeletal model employed in this study consists of a neural-driven forward dynamics model and static optimisation element [23]. This method enhances the joint torque estimation with static optimisation by adjusting the experimental EMG signals and synthesizing the muscle excitations that are not available. The objective function is defined in Equation (1) [23]:

(1) $$F=\alpha E_{trackMOM}+\beta E_{sumEXC}+\gamma E_{trackEMG}$$

where $E_{trackMOM}$ is the sum of squared differences between the estimated and the measured joint torques, $E_{sumEXC}$ is the sum of squared excitations for all the MTUs that are involved, including those with the experimental and the synthesized EMG envelopes, and $E_{trackEMG}$ is the sum of absolute differences between adjusted and experimental excitations. α, β and γ are weighting coefficients for each of the terms. Before computing the individual joint torques, the model parameters were tuned by minimising the difference between the torques predicted by the EMG-driven model and the measured torques, to increase the levels of subject specificity. Optimising ranges for individual parameters (Table 1) were assigned according to those reported previously [11,15]. 

As in [23], α was set as 1, and β and γ were determined such that $E_{trackMOM}$ and $E_{trackEMG}$ reached the minimum. Nineteen MTUs of the model were driven by the experimental EMG recordings from the muscles belonging to the corresponding innervation zones, as described in [15] (Table 2). Moments of hip, knee and ankle DoFs for each of the four trials were computed, with the measured data from the remaining three trials used for calibration. The estimation was executed in the EMG-assisted mode with the Calibrated EMG-Informed Neuromusculoskeletal modelling (CEINMS) toolbox [43]. 

### 2.4. Ground Reaction Force and Centre of Pressure Estimation

We applied artificial neural networks (ANNs) to estimate the three-dimensional (3D) GRF and COP in anterio-posterior (AP) and medio-lateral (ML) direction. Since it is known that the performance of ANN models can be strongly influenced by the input suitability [47], a subset of features was selected using Neighbourhood Component Analysis (NCA) prior to the estimation.

#### 2.4.1. Feature Selection by Neighbourhood Component Analysis

NCA is a nonparametric dimensionality reduction technique which learns the Mahalanobis distance used in the k-nearest neighbourhood classification algorithm [48]. NCA has been applied on kinematics, kinetics and physiological signals [49,50,51,52,53] and has been shown to outperform other conventional algorithms such as principle component analysis and reliefF [50,53]. This approach optimises the feature weights by minimising the objective function that measures the leave-one-out prediction loss over the training data. 

Analogous to the one-nearest-neighbour algorithm, NCA chooses point j as the neighbour of point i depending on the distance between the two points. The kernel function $k\left(z\right)=e^{\left(-\frac{z}{\sigma }\right)}$ is introduced such that the probability $p_{ij}$ is inversely proportional to the weighted distance $d_{w}$ where $w_{r}$ is the weight of feature r (Equations (2) and (3)).

(2) $$p_{ij}=\frac{k\left(d_{w}\left(x_{i,}x_{j}\right)\right)}{\sum_{j=1,j\neq i}^{n}k\left(d_{w}\left(x_{i,}x_{j}\right)\right)}$$

(3) $$d_{w}\left(x_{i,}x_{j}\right)=\overset{p}{\underset{r=1}{\sum }}w_{r}^{2}|x_{ir}-x_{jr}|$$

The average value of loss function is defined in Equation (4), in which $l\left(y_{i},y_{j}\right)$ is the mean absolute deviation between $y_{i}$ and $y_{j}$ in this study. A regularisation term with parameter λ is introduced in the objective function defined in Equation (5) to penalise the overfitting behaviour of the model [54].

(4) $$l_{i}=\overset{n}{\underset{j=1,j\neq i}{\sum }}p_{ij}l\left(y_{i},y_{j}\right)$$

(5) $$F\left(w\right)=\frac{1}{n}\overset{n}{\underset{i=1}{\sum }}l_{i}+\lambda \overset{p}{\underset{r=1}{\sum }}w_{r}^{2}$$

For estimating each of the parameters described in Section 2.4, this algorithm was applied to select a set of optimal features for each trial from the processed data, including joint angles, linear velocity and acceleration, angular velocity and acceleration, and EMG envelopes. Features with weights higher than 1% of the maximum values were selected.

#### 2.4.2. Estimation by Artificial Neural Network

Features selected by NCA were fed into separate ANN feedforward models for GRF and COP estimation in each direction. All the models were trained with one hidden layer [31,55]. The model weights and biases were updated through Levenberg–Marquardt backpropagation [56]. Hyperbolic tangent and linear activation transfer functions were employed for the hidden and output layers, respectively. Minimum gradient was used as stopping criteria to enhance the model generalisation. These designs align to those of previous studies for similar applications [31,55]. For every subject, each of the four trials was tested in turn. In each test case, the other three trials were regarded as training data, and were partitioned into five folds randomly for cross-validation. The model hyperparameters for each test were chosen through Bayesian optimisation by minimising the objective function defined in Equation (6):

(6) $$F_{obj}=\underset{k}{\max}(\sqrt{\frac{\sum_{n=1}^{N}{\left(y_{est,k}-y_{true,k}\right)}^{2}}{N}})$$

where N is the total number of test points, k is fold number, $y_{est,k}$ and $y_{true,k}$ are ANN output and measured values, respectively, for test point n at kth fold. The optimising ranges of hyperparameters are depicted in Table 3. 

### 2.5. Experimental Conditions and Model Performance Analysis

GRF was normalised to body weight (BW) and COP was analysed in millimetres. The ‘goodness-of-fit’ of GRF and COP estimates were assessed using the coefficient of determination $\left(R^{2}\right)$ and Root Mean Squared Error (RMSE) between the predicted and the measured values. 

**Figure 1:**
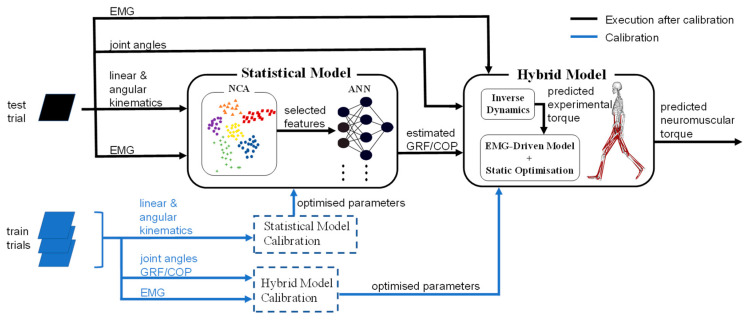
The schematic flowchart of the entire investigating paradigm for each subject. Prior to both GRF/COP estimation and torque computation, statistical and hybrid biomechanical models are calibrated using measured data from three train trials. After statistical model calibration, a set of optimal features as inputs to the ANN model are selected by NCA from the measured data of the test trial for the GRF/COP estimation. After the hybrid model calibration, GRF/COP estimates are fed into the biomechanical models (musculoskeletal and hybrid models) for joint torque prediction.

To investigate the overall and individual effects of estimated GRF and COP on both non-neural and neural based sagittal torque prediction, three experimental conditions for each type of model were established. As such, the following combinations of inputs were fed into both musculoskeletal and EMG-assessed models: (i) both estimated GRF and estimated COP (EGEC-M/EGEC-N), (ii) estimated GRF and measured COP, (EGMC-M/EGMC-N) and (iii) measured GRF and estimated COP (MGEC-M/MGEC-N). Abbreviations of conditions with suffix ‘M’ and ‘N’ denote musculoskeletal and neuromusculoskeletal models, respectively. All the musculotendon and model parameters were tuned with measured data for each subject. The same set of optimised parameters and EMG recordings were used for all the experimental conditions. The concept of running the entire investigating paradigm for each subject is illustrated in the schematic flowchart (Figure 1). The torque outputs from each model were normalised to the body mass, and compared to their respective measured values using metrics $R^{2}$ and RMSE. All trial lengths were normalised to 100 samples, with 0% and 100% corresponding to heel strike and toe-off, respectively. 

### 2.6. Statistical Analysis

Shapiro–Wilk, Levene’s and Mauchly’s tests were conducted to examine the data normality, variance homogeneity and sphericity, respectively. Whenever these assumptions were met, a three-way mixed ANOVA was applied to determine whether there was significant difference in both torque metrics for factors: subjects, experimental conditions and models. In case of nonexistence of significant interaction effects among the factors, only main effects were analysed. If significant interaction is present, focused repeated measures ANOVA was conducted for each level of the factor(s) concerned to determine the simple main effects. In addition, another repeated measures ANOVA was conducted with subject means to test on the same hypotheses. In case significant difference was detected, post hoc paired t-tests with Bonferroni correction were performed for pairwise comparison. If the test assumptions were violated, nonparametric within-subject and between-subject Friedman tests were conducted, followed by post hoc Dunn–Bonferroni tests. Significance level was set at *p* = 0.05. All analyses were carried out with IBM SPSS (version 27, SPSS Inc., Chicago, IL, USA).

## 3. Results

**Figure 2:**
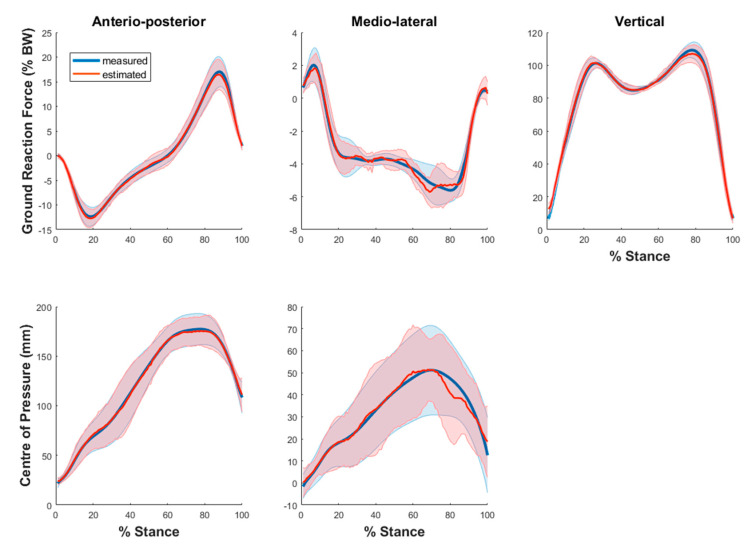
Measured (blue) and estimated (red) GRF (**top row**) and COP (**bottom row**) averaged across all the trials and subjects. GRF was normalised with respect to BW and COP illustrated in mm. Shaded area represents one standard deviation of the subject means.

**Figure 3:**
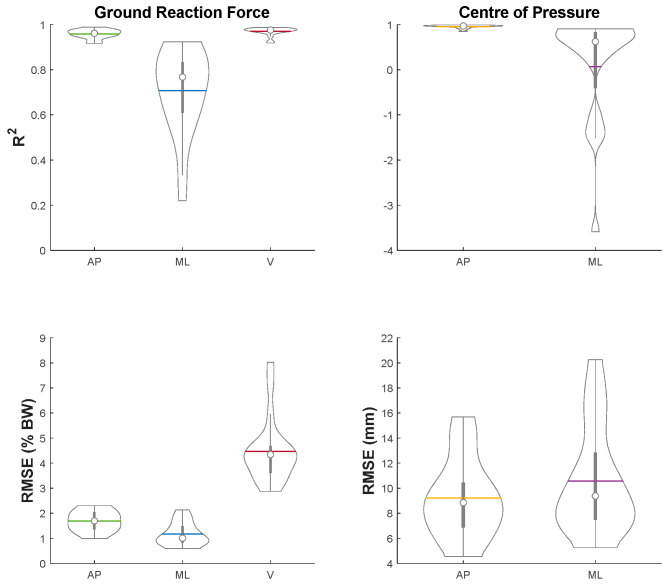
Distribution of $R^{2}$ (**top row**) and RMSE (**bottom row**) for GRF (**left column**) and COP (**right column**) for all the trials. RMSE for GRF and COP in % BW and in mm, respectively.

The average GRF and COP estimates from the ANN models across all the trials and subjects are depicted in Figure 2. The distribution of $R^{2}$ and RMSE for GRF and COP over all the trials are shown in Figure 3. The mean $R^{2}$ for vertical (V) GRF (0.97 ± 0.02) was the highest, followed by AP GRF (0.96 ± 0.02) and then ML GRF (0.71 ± 0.19). The $R^{2}$ for both GRF and COP in ML direction had a larger variability than those in AP and V direction. The mean $R^{2}$ for AP COP (0.96 ± 0.04) was considerably higher than ML COP (0.07 ± 1.19). The mean RMSE for V, AP and ML GRF were 4.5 ± 1.2, 1.7 ± 0.4 and 1.2 ± 1.2 % BW, while that for AP and ML COP were 9.2 ± 3.2 and 10.6 ± 4.3 mm, respectively.

**Figure 4:**
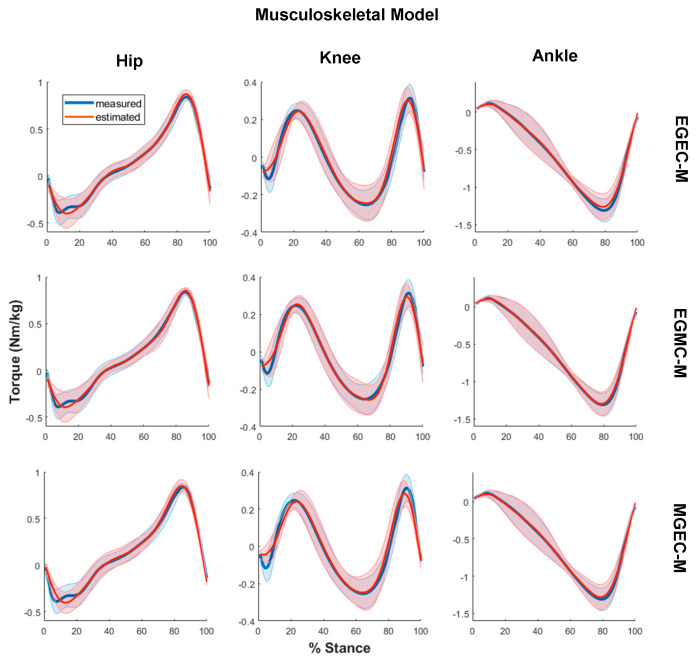
Measured (blue) and estimated (red) joint torques via musculoskeletal modelling for hip (**left column**), knee (**middle column**) and ankle (**right column**) for three conditions: estimated both GRF and COP (EGEC-M) (**top row**), estimated GRF only (EGMC-M) (**middle row**) and estimated COP only (MGEC-M) (**bottom row**) averaged across all the trials and subjects. Torques were normalised with respect to body mass. Data were plotted in percent of stance phase from heel strike to toe-off. Shaded area represents one standard deviation of the subject means.

**Figure 5:**
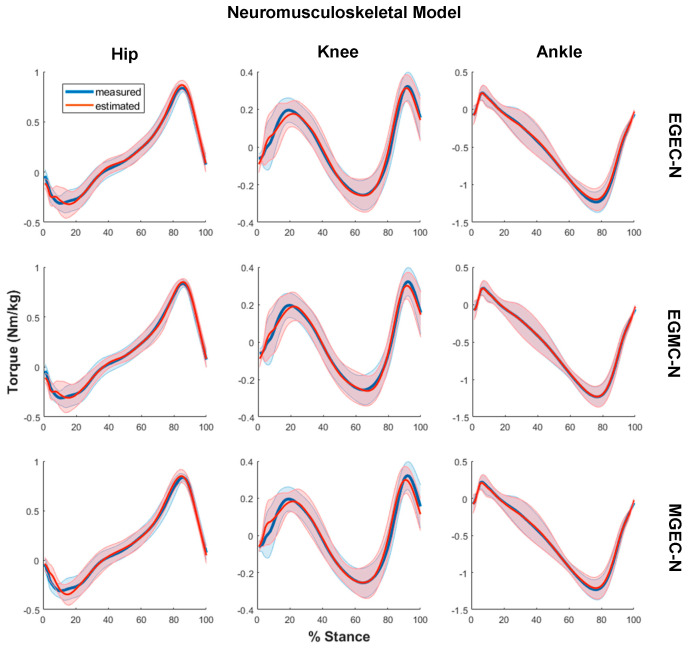
Measured (blue) and estimated (red) joint torques via neuromusculoskeletal modelling for hip (**left column**), knee (**middle column**) and ankle (**right column**) for three conditions: estimated both GRF and COP (EGEC-N) (**top row**), estimated GRF only (EGMC-N) (**middle row**) and estimated COP only (MGEC-N) (**bottom row**) averaged across all the trials and subjects. Torques were normalised with respect to body mass. Data were plotted in percent of stance phase from heel strike to toe-off. Shaded area represents one standard deviation of the subject means.

**Figure 6:**
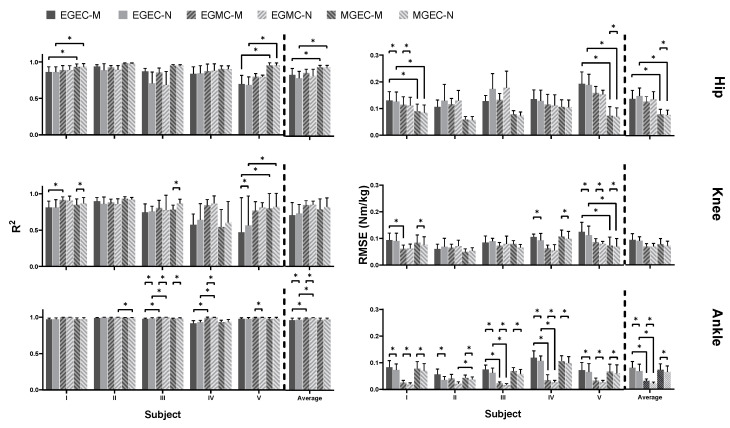
$R^{2}$ (**left column**) and RMSE (**right column**) for hip (**top row**), knee (**middle row**) and ankle (bottom row) for each of the following conditions: (i) estimated GRF and COP with muscu-loskeletal model (EGEC-M), (ii) estimated GRF and COP with neuromusculoskeletal model (EGEC-N), (iii) estimated GRF and measured COP with musculoskeletal model (EGMC-M), (iv) estimated GRF and measured COP with neuromusculoskeletal model (EGMC-N), (v) measured GRF and estimated COP with musculoskeletal model (MGEC-M), (vi) measured GRF and estimated COP with neuromusculoskeletal model (MGEC-N). Asterisk indicates statistically significant difference (*p* < 0.05).

The average joint torques computed via non-neural and neural models for all three experimental conditions, that is, (i) EGEC-M/EGEC-N, (ii) EGMC-M/EGMC-N and (iii) MGEC-M/MGEC-N, are illustrated in Figure 4 and Figure 5, respectively. The $R^{2}$ and RMSE of joint torques for all the conditions are shown in Figure 6, and Table 4 and Table 5. Statistical results showed that the data violated the assumptions of ANOVA and hence Friedman and Dunn–Bonferroni tests were conducted. 

In the between-model comparison, RMSE results indicated that only a few significant differences were detected for hip joint for each of the conditions (0.025 < *p* < 0.046). The ground reaction estimates affected the two models to a similar extent at the knee joint for EGEC and EGMC. However, there was significant difference at the knee joint for MGEC on three subjects (*p* = 0.046). For ankle joint, significant difference was observed for EGEC and MGEC on all the subjects, and for EGMC on four subjects (*p* = 0.046). Statistical results from $R^{2}$ showed the same trend. In summary, the two models tended to behave differently at the knee level when measured GRF and estimated COP were employed. This was also the case at the ankle regardless of conditions. 

In the between-condition comparison, $R^{2}$ and RMSE statistical results were consistent. At hip joint, both metrics between EGEC-M and MGEC-M, and EGEC-N and MGEC-N for Subject I and V as well as mean values were of significant difference (0.013 < *p* < 0.04). At knee joint, significant difference was observed between EGEC-M and MGEC-M, and between EGEC-N and MGEC-N for Subject V (*p* = 0.04), while between EGEC-M and EGMC-M for Subject I (*p* = 0.04). However, subject means revealed no significant difference for all the conditions. At ankle joint, significant difference was detected between EGEC-M and EGMC-M, and EGEC-N and EGMC-N for Subject III and IV as well as mean values for both measures (0.005 < *p* < 0.04). Furthermore, Subject II showed significant difference in both metrics between EGMC-N and MGEC-N (*p* = 0.04). Generally, MGEC-M and MGEC-N performed better than EGMC-M and EGMC-N at hip joint; however, they did contrariwise at the ankle.

## 4. Discussion

The primary focus of this study was to investigate the feasibility of predicting joint torques in sagittal plane via both musculoskeletal and neuromusculoskeletal models with reduced reliance on ground reaction measurements (force platforms). The motivation behind this effort comes from a desire to allow subject-specific high-fidelity model-based gait analysis to take place outside the laboratory. We applied machine learning algorithms to estimate GRF and COP, which were then used to compute the torques via inverse dynamics and hybrid neuromusculoskeletal modelling. Moreover, the study considered the individual importance of GRF and COP estimation accuracy for torque prediction. To this end, we introduced two additional experimental conditions: feeding models with either (i) estimated GRF and measured COP or (ii) measured GRF and estimated COP. We evaluated the accuracy of all three experimental conditions for both models with respect to the corresponding measured values, and the proportion of measured torque variance that was explained by the ground reaction estimates.

In the first part of this study, we estimated intrasubject 3D GRF as well as AP and ML COP using separate ANN models. The application of GRF/COP estimation using machine learning has been attempted by a number of studies [31,32,33,34,35,36,37,38,39]. Due to the different experimental setups, equipment used, amount and type of data available, and research goals, it is challenging to single out any of the previously applied machine learning algorithms and highlight the most appropriate method to establish the models for the current study. For this reason, we did not fully employ any of the aforementioned protocols, but instead we aligned the overall network design with the general good practices available in the literature, as detailed in Section 2.4.2. On the same grounds, it is challenging to make head-to-head comparison between our models and previously presented ones. However, we will still discuss the ‘goodness-of-fit’ of the ANN models as this is the investigating factor that affects the joint torques, which are of primary concern to the study.

Our average $R^{2}$ values for GRF, which were 0.96 for AP, 0.97 for V and 0.71 for ML directions, were all slightly higher than the previously reported values ranging from (0.92–0.94) for AP, (0.94–0.96) for V and (0.64–0.70) for ML directions [31,37,39]. Dorschky et al. [33] estimated GRF using combination of measured and simulated training data to solve the problem of data insufficiency for deep neural networks. This allowed them to obtain minimum average RMSE across subjects of 3.8% BW and 10.0% BW for AP and V GRF, respectively. In comparison, this study obtained the mean RMSEs for AP (1.7% BW) and V (4.5% BW) GRF. Regarding COP, the observed mean RMSE here for AP direction (9.2 mm) seemed to outperform that reported in Podobnik et al. [57] where the corresponding value of 14.9 mm was noted. On the other hand, our average RMSE for ML direction (10.6 mm) was higher than their reported value (9.0 mm). Moreover, in the current study, the error in AP was found smaller than that in ML direction, which contradicts the findings of previous study [57]. This could be, in part, due to the fact that we measured the COP using a different reference point. In summary, while it is difficult to directly compare the methods of GRF/COP estimation, our models seem to be of similar fit overall compared to those previously reported, thus making it suitable for supporting the outlined investigation on torque prediction. 

For the primary focus of this study, joint torques were predicted via both inverse dynamics and the application of the hybrid model, using the obtained GRF and/or COP estimates. Given the condition in which both GRF and COP were estimated (EGEC), the RMSE results exhibited significant differences between the two models at both knee and ankle for at least two subjects. Notwithstanding the close tracking of the hybrid model to inverse dynamics (i.e., no significant difference between the models was observed in [23]), the ground reaction estimates caused different effects on the two models for knee and ankle torque prediction in the current study. Among all three joints, hip was the most sensitive (RMSE = 0.14–0.15 Nm/kg) while ankle was the most resistant (RMSE = 0.069–0.081 Nm/kg) to the GRF/COP estimates, regardless of the model type. This finding corresponded to that of a related study [58], which conducted a sensitivity test to examine the effect of shear GRF on joint torques. This outcome could be explained by the fact that foot is the first body segment to be ‘solved’ in the process of inverse dynamics calculations. 

Lee and Park [39] estimated GRF and COP by relying on their biomechanical relationships to centre of mass of a three-dimensional spring walking model. The ‘goodness-of-fit’ reported here for hip ($R^{2}$ = 0.80–0.84) was slightly inferior to the one that they reported ($R^{2}$ = 0.85). However, this study achieved a better fit for knee ($R^{2}$ = 0.70–0.73) and ankle ($R^{2}$ = 0.97), compared to those obtained by them ($R^{2}$ = 0.30 for knee and $R^{2}$ = 0.83 for ankle). Ardestani et al. [40] estimated joint torques using a wavelet neural network with eight EMGs, AP and V GRF as inputs. Mundt et al. [41] predicted three-dimensional joint moments from kinematics and anthropometric measurements using recurrent ANN based on long short-term memory. The ankle torque fits presented in the current study were in agreement with those reported in the above two studies ($R^{2}$ = 0.96), whereas the fits for the knee and the hip were somewhat worse ($R^{2}$ = 0.70–0.84) compared to these works ($R^{2}$ = 0.86–0.96). These good estimation results reported previously reveal, to a certain extent, that the data-driven methods have the ability to capture relationships between EMG/kinematics and torques. While direct estimation using data-driven methods can bypass error accumulation rooted in GRF/COP estimation, it lacks transparency of torque formulation and the characterisation of personalised anatomy and neurophysiology.

In addition to these investigations, the individual effects of GRF and COP estimates on the joint torque prediction via the two models were considered. To this end, four additional conditions (two for each model): EGMC-M, EGMC-N, MGEC-M and MGEC-N were investigated. For hip, statistical results in two of the five subjects showed that there was significant difference between EGEC and MGEC, suggesting that the inaccuracies from the GRF affected torque estimates more than those from COP. This, as a trend, was also evident in the average results. Contrariwise, the ankle torques were more sensitive to inaccuracies of COP than those of GRF, as observed from two of the five individuals, as well as from the average outcomes. For knee, it could not be concluded whether GRF or COP inaccuracies had a larger effect on joint torques, as only one significant difference was found in each of the cases. Moreover, an obvious positive relationship was observed between $R^{2}$ of AP COP and that of ankle torque. To boost the ‘goodness-of-fit’ of GRF/COP, further research and comparison needs to be completed across a range of machine learning protocols with the same experimental setup and datasets. It is worth noting that plantar flexors, which are only responsible for ankle (and knee) flexion movements, were shown to have contributions to ML GRF [59]. Hence, this study accounted for GRF in all three directions, even though we only considered joint torques in sagittal plane.

Due to the exploratory nature of this study, we considered only five subjects, which limits investigations beyond those discussed here. Thus, a follow-up work on a larger subject pool would be valuable at this point. Another point to note is that this study used EMG recordings from eight muscles to run the neuromusculoskeletal model. While this corresponds to the setup of previous work [60], an investigation into the number of muscles to use for a good tradeoff between system portability and retrieved information should be considered. Moreover, experimental EMG signals that have proved poorly synthesized by the hybrid model [23] could be employed in future studies. To further leverage the advantage of underlying biomechanical models, future investigations involving gait impaired individuals could enhance the present approach through applications of imaging techniques (e.g., [61]). While this study mostly relied on measurements from a marker-based motion capture system, which is regarded as a benchmark, for estimation of ground reaction parameters, the relevant features used in the machine learning models could be obtained by inertial measurement units. Applying the presented approach with these wearable sensors, the portability of the entire experimental setup could be further enhanced.

This study has investigated the feasibility of sagittal joint torque prediction based on both non-neural and neural musculoskeletal hybrid models with statistical ground reaction estimates. This approach enables consideration of subject-specific anatomy and neurophysiology via hybrid modelling with limited use of force plates, which favours gait analysis in laboratory-free settings. The applied method results in the least torque estimation error in ankle, and the most in hip for both models. Furthermore, the individual impact of the GRF/COP estimates on joint torques has been examined. The outcome suggests future focus on improving different ground reaction estimates, which depends on the joint(s) of interest. This study helps pave the way towards gait analysis of this type in daily-life activities.

## Figures and Tables

**Table 1:** Optimising range of parameters for neuromusculoskeletal model.

Neuromusculoskeletal Model Parameter	Range	
	Minimum	Maximum
Tendon slack length	0.95	1.05
Optimal fibre length	0.975	1.025
Muscle strength coefficient	0.5	2
MTU activation filtering coefficients	−1	1
Shape factor	−5	0

**Table 2:** The recorded EMG and the MTU driven by the corresponding recorded EMG. MTU of the model depicted was abbreviated as in OpenSim.

Experimental EMG	MTU of the Model
Soleus	soleus
Tibialis anterior	tib_ant
Gastrocnemius medialis	med_gas, lat_gas
Rectus femoris	rect_fem
Vastus medialis	vas_lat, vas_med, vas_int
Semitendinosus	semimem, semiten
Gluteus maximus	glut_max1, glut_max2, glut_max3
Gluteus medius	glut_med1, glut_med2, glut_med3, glut_min1, glut_min2, glut_min3

**Table 3:** The optimizing range for ANN model parameter.

ANN Model Parameter	Range	
	Minimum	Maximum
Neuron number	2	20
Learning rate	0.0005	1
Epochs	500	1000

**Table 4:** $R^{2}$ of joint torques averaged across all the trials and subjects for all the conditions: (i) estimated GRF and COP with musculoskeletal model (EGEC-M), (ii) estimated GRF and COP with neuromusculoskeletal model (EGEC-N), (iii) estimated GRF and measured COP with musculoskeletal model (EGMC-M), (iv) estimated GRF and measured COP with neuromusculoskeletal model (EGMC-N), (v) measured GRF and estimated COP with musculoskeletal model (MGEC-M), (vi) measured GRF and estimated COP with neuromusculoskeletal model (MGEC-N).

Condition		Joint	
	Hip	Knee	Ankle
EGEC-M	0.84 ± 0.089	0.70 ± 0.17	0.97 ± 0.029
EGEC-N	0.80 ± 0.094	0.73 ± 0.12	0.97 ± 0.024
EGMC-M	0.87 ± 0.048	0.84 ± 0.059	0.99 ± 0.0027
EGMC-N	0.83 ± 0.090	0.84 ± 0.051	1.0 ± 0.0013
MGEC-M	0.95 ± 0.028	0.78 ± 0.14	0.97 ± 0.023
MGEC-N	0.94 ± 0.028	0.82 ± 0.12	0.97 ± 0.021

**Table 5:** RMSE of joint torques averaged across all the trials and subjects for all the conditions: (i) estimated GRF and COP with musculoskeletal model (EGEC-M), (ii) estimated GRF and COP with neuromusculoskeletal model (EGEC-N), (iii) estimated GRF and measured COP with musculoskeletal model (EGMC-M), (iv) estimated GRF and measured COP with neuromusculoskeletal model (EGMC-N), (v) measured GRF and estimated COP with musculoskeletal model (MGEC-M), (vi) measured GRF and estimated COP with neuromusculoskeletal model (MGEC-N). The unit for RMSE is Nm/kg.

Condition	Joint		
	Hip	Knee	Ankle
EGEC-M	0.14 ± 0.032	0.094 ± 0.024	0.081 ± 0.023
EGEC-N	0.15 ± 0.030	0.091 ± 0.016	0.069 ± 0.026
EGMC-M	0.13 ± 0.019	0.070 ± 0.010	0.032 ± 0.0073
EGMC-N	0.14 ± 0.029	0.070 ± 0.011	0.022 ± 0.0045
MGEC-M	0.081 ± 0.018	0.079 ± 0.021	0.073 ± 0.029
MGEC-N	0.078 ± 0.018	0.073 ± 0.017	0.065 ± 0.023

## Data Availability

This research has been conducted with previously published dataset [42].
